# Evaluation of Neuroprotective and Neuroregenerative Potential of NeuroAiD™ II(MLC901) in a Rat Model of Kainic Acid-Induced Spinal Cord Injury

**DOI:** 10.1007/s12035-025-05064-4

**Published:** 2025-06-06

**Authors:** Anam Anjum, Muhammad Dain Yazid, Muhammad Fauzi Daud, Jalilah Idris, Angela Min Hwei Ng, Amaramalar Selvi Naicker, Ohnmar Htwe Rashidah Ismail, Ramesh Kumar Athi Kumar, Yogeswaran Lokanathan

**Affiliations:** 1 Department of Tissue Engineering and Regenerative Medicine, Faculty of Medicine, Universiti Kebangsaan Malaysia, 56000 Kuala Lumpur, Cheras Malaysia; 2 University of Maryland School of Medicine, Baltimore, MD 21201 USA; 3 Institute of Medical Science Technology, Universiti Kuala Lumpur Malaysia, 43000 Kajang, Selangor Malaysia; 4 Department of Orthopaedics & Traumatology, Faculty of Medicine, Universiti Kebangsaan Malaysia, 56000 CherasKuala Lumpur, Malaysia; 5 Department of Orthopaedics and Traumatology, Faculty of Medicine, Universiti Sultan Zainal Abidin (UniSZA), 21300 Kuala Terengganu, Malaysia; 6 Department of Surgery, Hospital Canselor Tuanku Muhriz, Universiti Kebangsaan Malaysia, 56000 Kuala Lumpur, Cheras Malaysia; 7 Advance Bioactive Materials-Cells UKM Research Group, Universiti Kebangsaan Malaysia, 43600 Bangi, Selangor Malaysia

**Keywords:** Spinal cord injury, Glutamate receptors agonist, Kainic acid excitotoxicity, Nerve regeneration, NeuroAiD II (MLC 901), GAP43, GFAP

## Abstract

Excitotoxic damage caused by high extracellular levels of glutamate in the spinal cord results in neuronal loss and severe locomotor impairment. This study investigates the efficacy of NeuroAiD II (MLC901), an herbal formulation, in promoting nerve regeneration following spinal cord injury (SCI) induced by kainic acid (KA). KA, a potent glutamate receptor agonist, causes excitotoxic damage in the spinal cord, leading to neuronal loss and locomotor impairment. To explore the potential of MLC901, KA-injured rats were treated with MLC901, and nerve regeneration was evaluated using various techniques. In this study, KA was administered intrathecally between the T12 and T13 vertebrae in rats, resulting in incomplete paraplegia. MLC901 was then tested for its neuro-regenerative potential. Various assessments were conducted to evaluate the effects of MLC901 treatment, including behavioral, electrophysiological, and histopathological analyses. Behavioral tests, such as the Basso, Beattie, and Bresnahan (BBB) open field test, running wheel, grid walk, inverted grid, and sensory tests, showed significant improvements in locomotor activity in treated rats. Electrophysiological recordings indicated that, while KA injection caused reduced amplitude and delayed latency, MLC901 treatment helped restore lost connections on days 14 and 28. Histopathological and immunohistochemical analyses also revealed improved tissue integrity and neuron survival. The study concludes that MLC901 significantly enhances locomotor recovery, somatosensory evoked potentials, and tissue preservation following SCI. These findings suggest that MLC901 holds promise as a neuro-regenerative therapy for spinal cord injuries.

**Supplementary Information:**

The online version contains supplementary material available at 10.1007/s12035-025-05064-4.

## Introduction

Spinal cord injury (SCI) is a devastating condition with no effective treatment currently available [1]. Globally, the annual incidence of SCI varies widely, ranging from 11.5 to 53.4 cases per million inhabitants. This variation is likely due to differences in reporting practices and regional factors. For instance, in North America, the incidence is about 39 cases per million, while in Western Europe, it is 16 per million. The estimated number of people living with SCI worldwide ranges from 236 to 4187 per million, with significant regional disparities [2]. SCI imposes a substantial financial burden on individuals and healthcare systems. Recent estimates indicate that the average annual expenses for a person with SCI vary significantly based on the severity and level of the injury. A systematic review reported that the first-year costs post-SCI range from $32,240 to $1,156,400, with annual expenses in subsequent years between $4490 and $251,450 [3]. Another analysis estimated lifetime expenditures per individual with SCI between $700,000 and $2.5 million, influenced by factors such as age at injury and neurological level [4]. Conventional models of SCI employ various mechanical methods, such as contusion, compression, transection, and dislocation, to investigate SCI pathophysiology [1]. However, chemical-based methods for inducing SCI are gaining popularity due to their ease of use and ability to produce specific degenerative pathways [5].

KA is a potent agonist of ionotropic glutamate receptors and is widely used to study excitation-induced neuronal apoptosis both in vivo and in vitro [6, 7]. Excitotoxicity, a process of neuronal cell death caused by excessive activation of glutamate receptors, plays a crucial role in various nervous system disorders, including brain and spinal ischemia, trauma, and neurodegenerative diseases [5, 7]. L-glutamate, a major excitatory neurotransmitter, is involved in synaptic neurotransmission, neuronal migration, and excitability [8]. Overactivation of glutamate receptors by KA increases intracellular calcium ion influx, producing reactive oxygen species (ROS) and reactive nitrogen species (RNS), which in turn causes neuronal death through oxidative stress and ATPase activation [9]. KA initially damages neuronal membranes, leading to a disruption in ion gradients and a loss of cellular homeostasis. This results in excessive release of glutamate, the primary excitatory neurotransmitter in the central nervous system. Elevated extracellular glutamate levels overstimulate glutamate receptors, particularly NMDA receptors, leading to an influx of calcium ions into neurons. This calcium overload triggers a cascade of events, including mitochondrial dysfunction, activation of destructive enzymes, oxidative stress, and the initiation of apoptosis [10]. Our previous protocol detailed a method for inducing SCI in SD rats using KA [5].

Many natural compounds, such as *Centella asiatica* (also known as Gotu Kola) [11], curcumin (from turmeric) [12], Ginkgo biloba [13], and *Bacopa monnieri* [14], show promising neuroprotective properties, helping to reduce inflammation and oxidative damage, two factors involved in many neurological conditions. MLC901 is a herbal formulation that contains nine components, i.e., Radix Astragali, Radix Salviae Miltiorrhizae, Radix Paeoniae Rubra, Rhizoma Chuanxiong, Radix Angelicae Sinensis, Carthamus Tinctorius, Prunus Persica, Radix Polygalae, and Rhizoma Acori Tatarinowii [15, 16]. The key active ingredients in MLC901 include tetramethylpyrazine (from Rhizoma Chuanxiong), ferulic acid (from Radix Angelicae Sinensis and Rhizoma Chuanxiong), ligustilide, butylidenephtalide, astralagoside IV, salvianolic acid B, and tanshinone IIB. These compounds are well known for their neurobeneficial and anti-inflammatory properties [15]. MLC901 helps reduce excessive glutamate release and calcium influx into neurons, preventing excitotoxic cell death. This is particularly important in SCI models, where glutamate-induced excitotoxicity is a major driver of secondary neuronal loss. MLC901 exerts neuroprotective effects through multiple mechanisms; it reduces neuroinflammation by modulating pro-inflammatory and anti-inflammatory cytokines, enhances neurogenesis by promoting the proliferation of neural progenitor cells [16], and protects against neuroapoptosis by regulating key apoptotic markers like Caspase-3 [17]. Additionally, MLC901 supports neuronal survival and function through angiogenesis, which improves blood flow and nutrient supply to the nervous tissue [18]. These combined actions help maintain the integrity and function of the nervous system under stress conditions like injury or disease.

Previous studies have shown that MLC901 enhances neural circuit restoration, cell proliferation, and stimulation of axonal and dendritic circuits following traumatic brain injury (TBI) and SCI [16, 19, 20]. The nerve regenerative potential of MLC901 has shown promise in improving outcomes after SCI [16, 21, 22]. An in vitro model using KA to induce excitotoxic cell death in differentiated motor neurons (dNSC-34) and the efficacy of MLC901 in promoting neuronal cell survival was also evaluated previously [7]. This study aims to assess the effectiveness of MLC901 in promoting locomotor, electrophysiological, neurological, and histological improvements following KA-induced SCI in SD rats. By comparing treated rats with untreated controls, the study aims to enhance understanding of disease progression, support the development of new interventional strategies for chemical SCI models, and demonstrate the neuro-regenerative and neuroprotective potential of MLC901 as a novel therapeutic approach.

## Results

### MLC901 Treatment Improves Functional Recovery and Hindlimb Movement After KA Administration

**Fig. 1:**
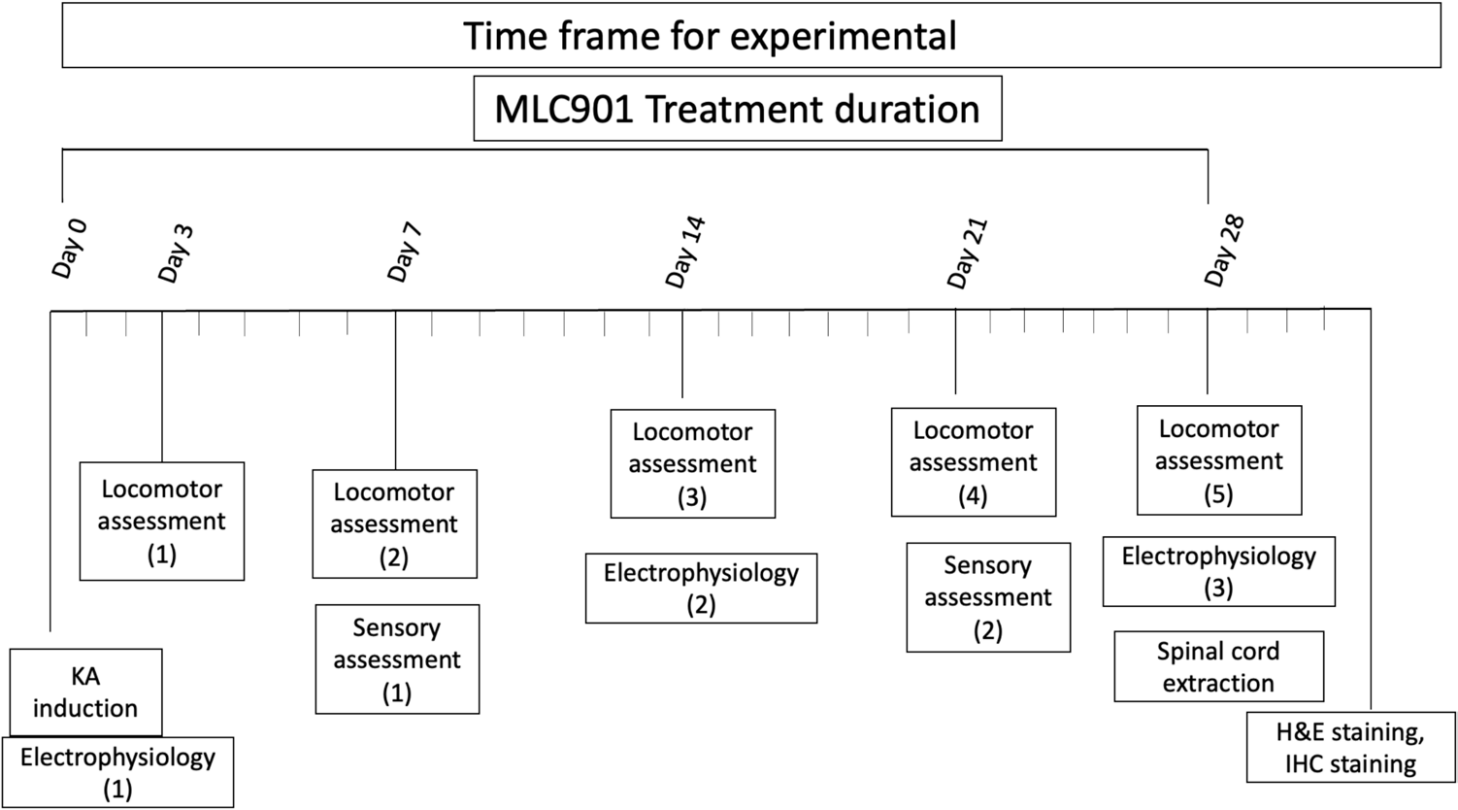
Experimental timeline overview. This timeline provides a structured approach to evaluating the effects of KA injury and MLC901 treatment, integrating assessments of motor function, electrophysiological responses, sensory perception, and histopathological changes over the experimental period

The study included the locomotor, sensory, and electrophysiological assessment, and the timeline of each assessment is shown in Fig. 1, with the study flow chart in Supplementary Figure S1. After KA injury, the rats exhibited complete paraplegia, with no movement in the hind limbs or tail and urination dysfunction, though defecation was unaffected. Urinary function improved by day 7 in both treated (T) and untreated (UT) rats, it was observed that urine volume per void was lower on day 3, showing some urine remained in the bladder, which was fully resolved at day 7 (Figure S2).

**Fig. 2:**
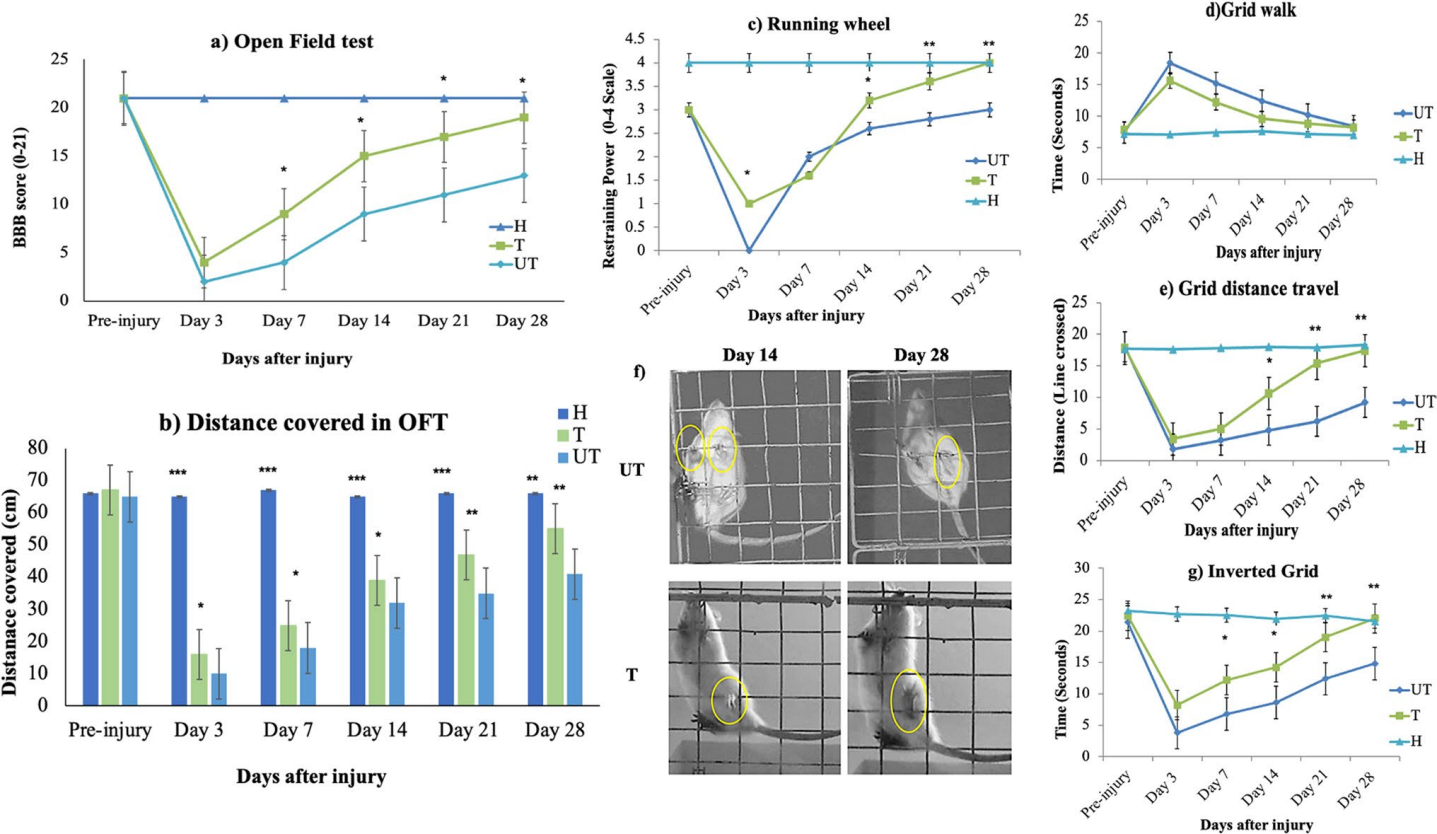
Effect of MLC901 on the locomotor and gait analysis after KA injury in rats. H, healthy rats; T, treated rats with 10 mg/kg/day of MLC901; UT, untreated rats. **a** BBB scores: The BBB scores for healthy (H) and injury rats (UT and T) were assessed pre-injury and post-injury. Pre-injury and H rats both received a BBB score of 21. After injury, the BBB scores for UT rats were significantly lower on days 7, 14, 21, and 28 compared to the T group (*p* < 0.05*), with *n* = 5 for each group. Data were analyzed using one-way analysis of variance (ANOVA) followed by post-hoc tests following Dunnett’s analysis. **b** Distance covered: The distance covered by H, UT, and T rats during a 5-min test was significantly reduced on days 3, 7, and 14 for UT rats compared to the T group (*p* < 0.05*). On days 21 and 28, the distance covered by UT rats remained significantly lower compared to T rats (*p* < 0.01**). The H group traveled a significantly greater distance on each day of assessment compared to both UT and T rats (*p* < 0.001***), with *n* = 5 for each group. Data were analyzed using one-way analysis of variance (ANOVA) followed by post-hoc tests following Dunnett’s analysis. Other locomotor assessment: Various locomotor tests were performed, including the **c** running wheel, **d** grid walk test, **e** grid distance travel, **f** foot placement faults, and **g** inverted grid. The T rats showed statistically significant improvements on days 7, 14, 21, and 28 post-injuries compared with UT rats (*p* < 0.01** and *p* < 0.05*). The number of foot faults and missed steps were also recorded, highlighting the improved performance of T rats compared to UT rats (*n* = 5). Data were analyzed using one-way analysis of variance (ANOVA) followed by post-hoc tests following Dunnett’s analysis

Retraction to the injury began to show after the first week in both the T and UT groups. The Basso, Beattie, and Bresnahan (BBB) scale was performed on days 7, 14, 21, and 28, the result indicated that the T group exhibited higher BBB scores than the UT group (Fig. 2a and b). In the T group, slight hind-limb movement was observed from the first week and became more pronounced by the third week. In contrast, the UT group only showed minimal coordinated movements after 14 days, with no significant improvement until day 28. By day 28, the T rats demonstrated more coordinated hind limb activity compared to the UT group, which showed less improvement (Fig. 2a). The BBB score sub-score revealed that T rats scored significantly better in all three subcategories compared to the UT rats by day 28 (*p* > 0.05*). Specifically, the T rats showed significant improvements in jaw movements, paw placement, and toe clearance compared to the UT rats, with statistical significance (*p* < 0.01 and *p* < 0.05) as illustrated in Figures S3a, b, and c.

The distance covered by the rats in the open field test is shown in Fig. 2b. UT rats exhibited a significantly reduced distance covered on days 3, 7, and 14 compared to the T group (*p* < 0.05). Specifically, the mean distances covered were at day 3; UT rats covered 10.5 ± 0.71 cm, while T rats covered 17.5 ± 0.76 cm (*p* < 0.05*). On day 7, UT rats covered 17.5 ± 0.71 cm, while T rats covered 25 ± 2.71 cm (*p* < 0.05*). While at days 14 and 21, UT rats covered 32.5 ± 2.12 cm and 35 ± 2.82 cm, while T rats covered 39.2 ± 3.53 cm and 47 ± 1.12 cm. On day 28, the UT rats covered 41 ± 2.21 cm, while the T rats covered 55 ± 2.43 cm. On days 21 and 28, the distance covered by T rats was significantly higher (*p* < 0.01**) compared to UT rats. The reduced distance in UT rats was attributed to slower improvement in coordination compared to the MLC901 treatment group (Fig. 2a, b).

Running wheel performance was assessed pre-injury and post-injury, with both UT and T rats showing a complete loss of restraining power immediately after injury. Both groups demonstrated significantly reduced performance compared to healthy (H) rats (*p* < 0.001***). However, on days 3 and 14, T rats exhibited significant improvement (*p* < 0.05*), with even greater improvement on days 21 and 28 (*p* < 0.01**) compared to UT rats (Fig. 2c). Both injured groups (UT and T) initially showed significant deficits in grid-holding strength and walking ability with high foot placement faults observed (Fig. 2d, e, and f). However, with continued treatment, T rats demonstrated substantial improvement (*p* < 0.05* and *p* < 0.01**) in both holding time and grid distance traveled compared to UT rats. Significant improvements were observed on days 7, 14, 21, and 28 (Fig. 2d, e, f, and g).

### MLC901 Treatment Improves the Sensory Functions Following KA Injury

**Fig. 3:**
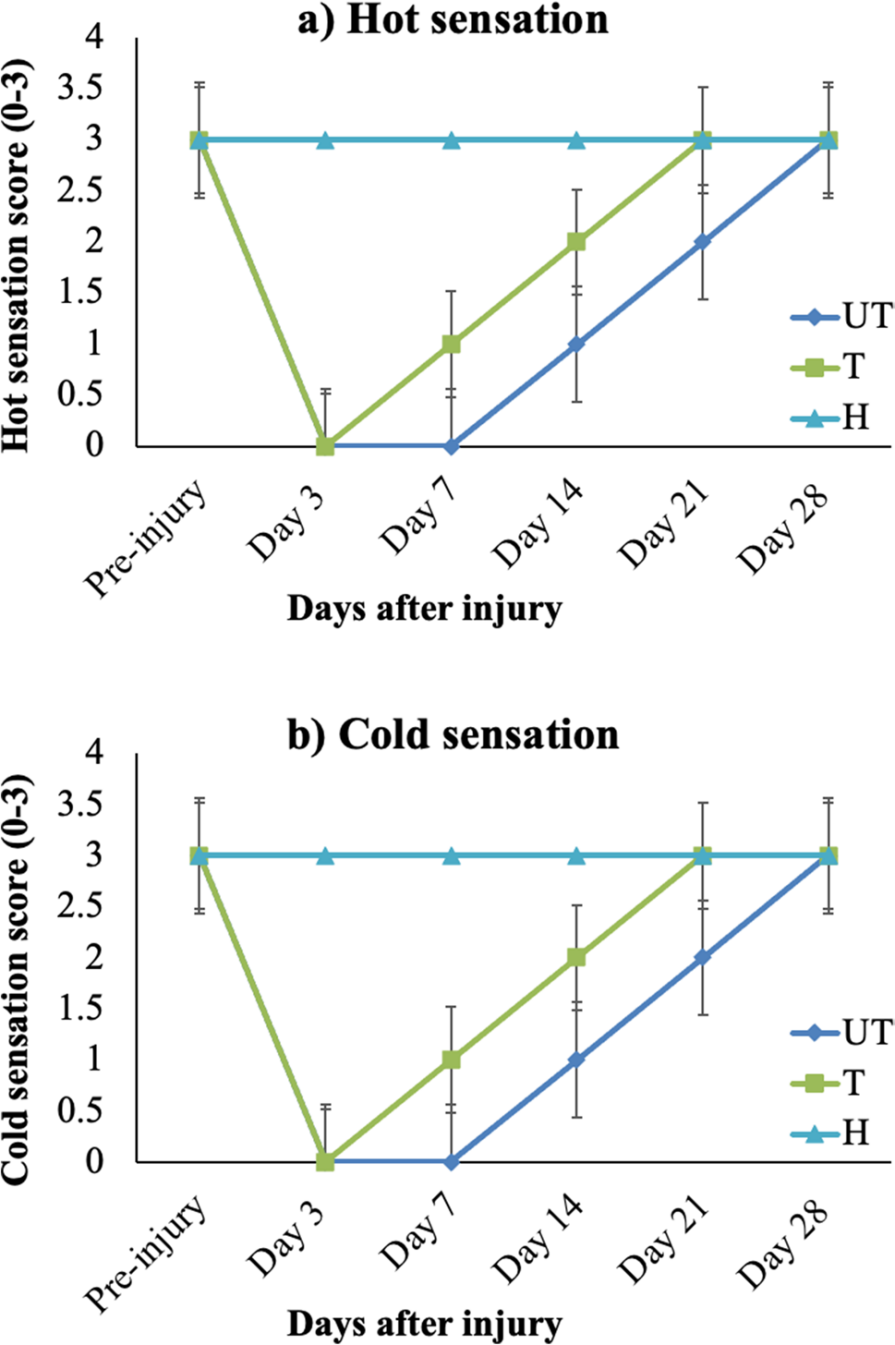
MLC901 improves sensory function test after KA SCI in rats. H, healthy rats; T, treated rats with 10 mg/kg/day of MLC901; UT, untreated rats. **a** Hot and **b** cold sensory function test in H, untreated (UT), and MLC901-treated (T) rats following kainic acid (KA) injury. The response scores for these tests indicated the following: Day 3: Both UT and T rats exhibited no response to hot and cold stimuli. Day 7: UT rats scored 0, showing no response, while T rats showed a slow withdrawal and scored 1. Days 14, 21, and 28: T rats consistently scored better than UT rats in both hot and cold sensation tests, indicating improved sensory function over time. The improved scores in T rats compared to UT rats across all assessed days (3, 7, 14, 21, and 28) demonstrate the positive impact of MLC901 treatment on sensory nerve recovery post-injury, but no significance was observed. Data was represented as mean with SEM (standard error of the mean). Data were analyzed using one-way variance analysis (ANOVA) followed by post-hoc tests following Dunnett’s analysis, *n* = 5

Sensory functions were assessed on days 7 and 21 following the KA injury. On day 3 post-injury, both UT and T rats exhibited no response to thermal stimulus. By day 7, UT rats consistently scored 0 by both observers, indicating a complete lack of response, while T rats demonstrated a slow withdrawal response and scored 1 (Fig. 3a, b). In contrast, pre-injury evaluations showed that all rats, including the H group, received a score of 3, indicating normal sensory function with quick withdrawal. The H group maintained a score of 3 throughout the experiment, reflecting normal sensory response with rapid withdrawal. Notably, the latency of the withdrawal response (the time between stimulation and the withdrawal of the hind paw) was shorter in the H group compared to the KA-injured rats. Among the injured rats, T rats displayed a shorter latency in the withdrawal response compared to UT rats (Fig. 3a, b).

### Impact of MLC901 Treatment on Electrophysiological Assessment

**Fig. 4:**
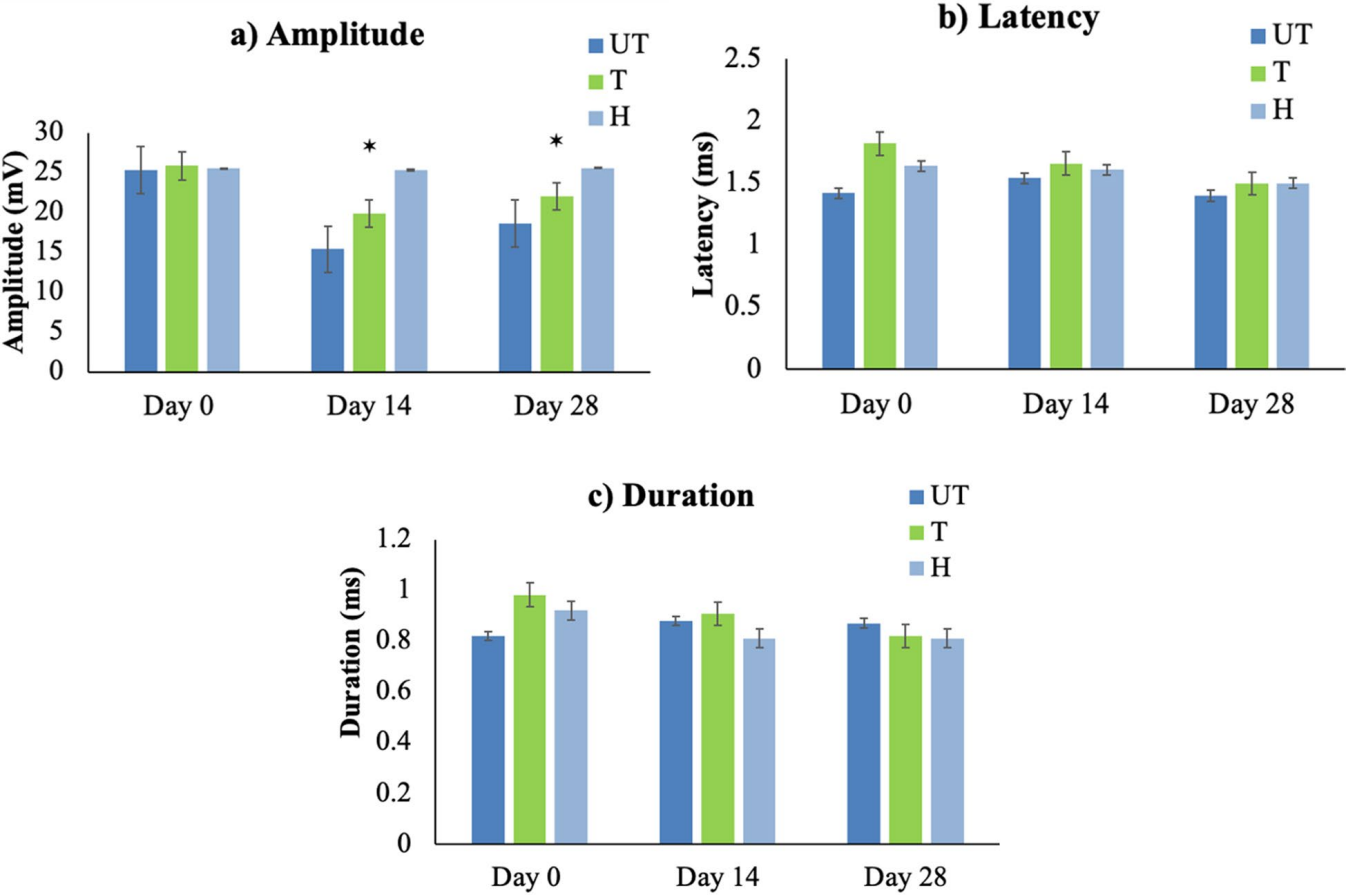
Somatosensory evoked potentials (SEPs) analysis. H, healthy rats; T, treated rats with 10 mg/kg/day of MLC901; UT, untreated rats. **a** Amplitude of SEPs in untreated (UT), MLC901-treated (T), and healthy (H) rats at pre-injury, day 14, and day 28 after injury. Statistically significant differences (*p* < 0.05*) in amplitude were observed between UT and T groups on days 14 and 28. **b** Duration of SEPs in UT, T, and H rats at pre-injury, day 14, and day 28. The duration reflects nerve conduction velocity, with the UT group showing significantly longer durations compared to the T group. **c** Latency of SEPs in UT, T, and H rats at pre-injury, days 14 and 28. The latency was significantly increased in UT rats compared to T rats after day 14. Data are presented as mean ± SD and analyzed using one-way variance analysis (ANOVA) followed by post-hoc tests following Dunnett’s analysis

Somatosensory evoked potential (SEPs) waveforms were absent in both UT and T rat groups, with no waveform detected even 30 min post-injury, while H rats, however, showed no significant changes in SEPs over time (Fig. 4a), the representative SEPs waveforms of different groups are shown in Figure S4.

### (i) Waveform analysis and amplitude

The amplitude decreased in both injured groups compared to pre-injury and H groups on days 14 and 28. Specifically, the mean onset amplitudes in T rats showed an amplitude of 19.56 ± 1.52 mV on day 14 and 22.3 ± 0.81 mV on day 28. In contrast, UT rats showed a significant decrease (*p* < 0.05*) in the mean onset amplitudes with 15.07 ± 1.72 mV on day 14 and 18.95 ± 1.43 mV on day 28. While H rats showed consistent amplitudes of 25.63 ± 0.81 mV on day 0, 25.67 ± 0.50 mV on day 14, and 26.02 ± 1.14 mV on day 28 (Fig. 4a). The UT-injured group exhibited a statistically significant reduction in amplitude compared to the T group on days 14 and 28 (Fig. 4a).

### (ii) Duration

Nerve conduction duration was significantly longer in the UT group compared to the T group. On day 14, the duration was 1.42 ± 0.17 ms in the UT group versus 1.32 ± 0.11 ms in the T groups (Fig. 4c). The increased duration in UT rats indicates slower nerve conduction and delayed signal transmission.

### (iii) Latency

There was no change observed in latency between UT rats and T rats after day 14 and day 28. While no waveform was observed immediately post-injury, by day 14, UT rats showed marked motor dysfunction, as indicated by reduced amplitude and increased duration, in contrast to the T group (Fig. 4).

### MLC901 Treatment Promotes the Survival of Neurons and Improves Tissue Density

**Fig. 5:**
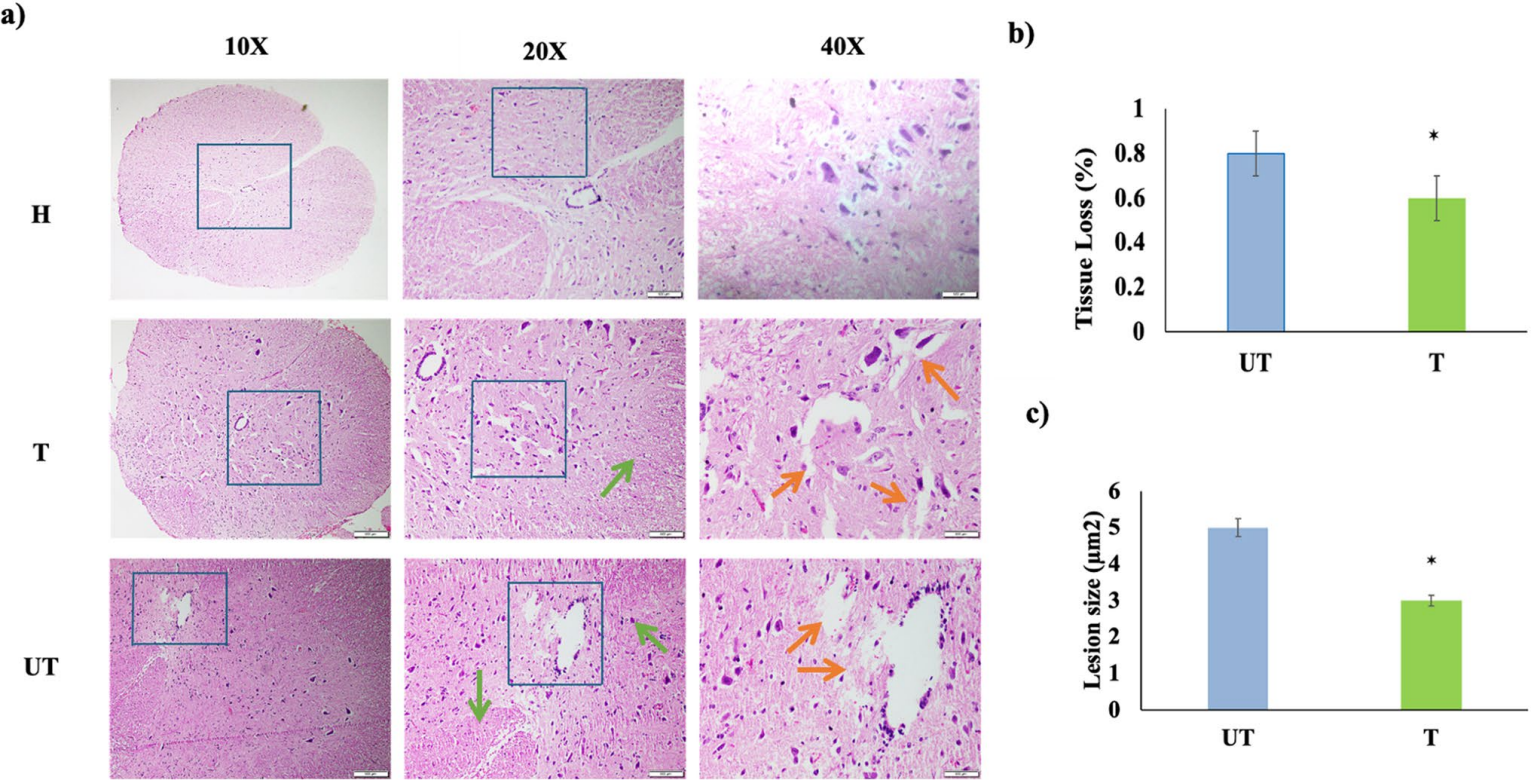
Histopathological analysis of spinal cord Sects. 28 days post-injury with MLC901 treatment. H, healthy rats; T, treated rats with 10 mg/kg/day of MLC901; UT, untreated rats. **a** The transverse sections of the spinal cord from healthy (H), untreated (UT), and MLC901-treated (T) rats, 28 days after kainic acid (KA) injury. The images illustrate hematoxylin and eosin (H&E) staining, revealing significant pathological spinal cord changes. The presence of erythrocytes and neutrophils is evident, indicating inflammation at the injury site in both UT and T groups indicating neuroinflammation, *n* = 5. **b** Tissue loss and hemorrhage foci: The darker stained areas indicate hemorrhagic foci, which appear pinkish. Pockets of erythrocytes are marked with green arrows, the percentage of tissue loss was calculated by a relative change to a baseline value, with the baseline being set as 0% tissue loss. **c** Lesion size: Progressive necrosis and cavitation are shown with orange arrows, highlighting the appearance of lesions in both UT and T sections. The lesion size was calculated using ImageJ software. Magnifications used are 10 ×, 20 ×, and 40 ×, with a scale bar of 500 µm. Each magnification level reveals the extent of tissue damage, with the UT group showing more severe hemorrhage and inflammation compared to the T group. Data are presented as *n* = 5. Data are presented as mean ± SD and were analyzed using one-way analysis of variance (ANOVA) followed by the post-hoc tests following Dunnett’s analysis

Hematoxylin and eosin (H&E) staining revealed severe lesions at the KA injection site, with the most significant damage occurring in the grey matter. Macroscopic evaluation showed hemorrhage, tissue destruction, and necrosis at the lesion site, with erythrocytes and neutrophils present in both UT and T rats. Albumin extravasation, indicating increased blood–brain barrier permeability due to glutamate excitotoxicity, was observed in both groups but was more pronounced in UT rats. The T rats exhibited smaller hemorrhagic foci, reduced cavity size, and less albumin leakage compared to UT rats (Fig. 5). Erythrocytes and neutrophils were found in abundance at the primary lesion site in both UT and T rats (Fig. 5a, b, c). Figure 5b represents % of tissue loss; the % of tissue loss was calculated by measuring relative tissue loss compared to a baseline value, where the baseline is set as 0% tissue loss. Figure 5c shows the lesion size (µm2), calculated using ImageJ.

### MLC901 Treatment Increases the Expression of GAP43 and Decreases the Expression of GFAP

**Fig. 6:**
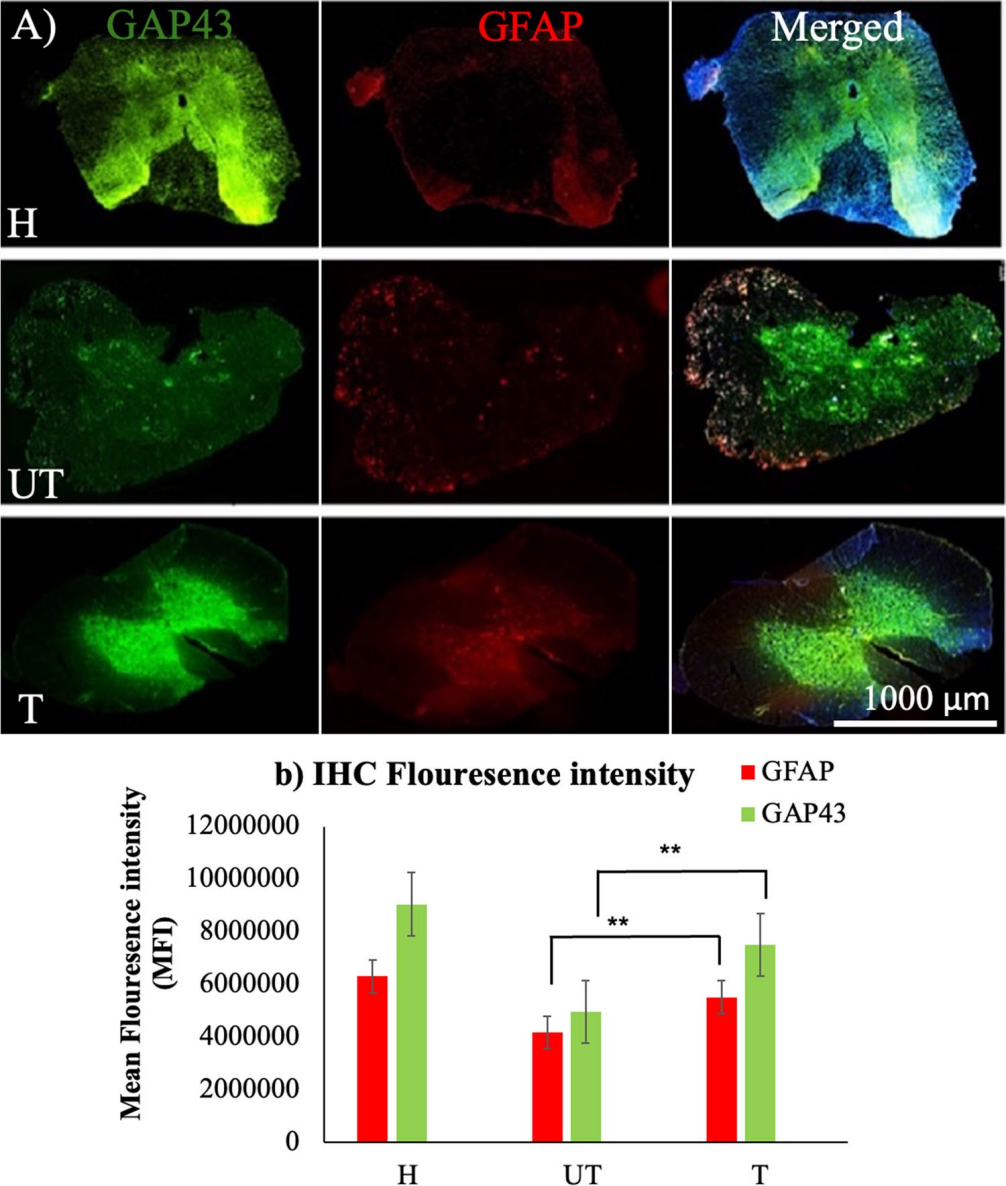
Immunohistochemical analysis of GAP-43, GFAP, and DAPI expression after MLC901 treatment in KA injured rats. H, healthy rats; T, treated rats with 10 mg/kg/day of MLC901; UT, untreated rats. **a** Transverse spinal cord sections from healthy (H), untreated (UT), and MLC901-treated (T) rats were stained for GAP-43 (green fluorescence), GFAP (red fluorescence), and DAPI (blue fluorescence). GAP-43 is a marker for motor neurons, GFAP is a marker for astrocytes and glial cells, and DAPI stains nuclei. In the H group, GAP-43/GFAP staining indicates abundant motor neurons with healthy neuronal fiber presence. **b** Fluorescence Intensity: Quantitative analysis of fluorescence intensity for GAP-43, GFAP, and DAPI in the H, UT, and T groups. The fluorescence intensity was measured to assess the levels of GAP-43 and GFAP expression. The UT group shows significantly lower fluorescence intensity for GAP-43 and GFAP compared to the T group, indicating greater neuronal and astrocyte damage. The T group shows comparatively higher levels of both GAP-43 and GFAP, reflecting partial recovery and preservation. Images were captured using a confocal microscope at 10 × magnification, with a scale bar of 1000 µm. Fluorescence intensity was analyzed and compared using ImageJ software. Data represented as mean ± SD were analyzed using one-way analysis of variance (ANOVA) followed by the post-hoc tests following Dunnett’s analysis, *n* = 5

Immunohistochemical staining of spinal cord sections focused on the expression of GAP-43 and GFAP. The protein expressions of GAP43 were used to evaluate neural regeneration. GAP-43 expression was significantly downregulated in the UT group compared to the T group (Fig. 6a, b). These results indicated that MLC901 had a positive effect on the survival and regeneration of axons. In contrast, the UT group displayed lower GAP43 expression, indicating greater neuronal loss compared to the T group (Fig. 6b). The co-staining of GAP-43/DAPI, and GFAP/DAPI expression in H, UT, and T groups are presented in Figure S5.

The GFAP expression was used as a marker to evaluate the astrocyte response to injury. The T group showed higher GFAP expression, indicative of astrocyte activation and reactive response to the injury. Reactive astrocytes play an important role in tissue repair after injury by forming a glial scar, which can help protect surrounding tissues. UT rats exhibited less astrocyte activation expression than the T rats (Fig. 6b). Confocal images reveal significant differences in the expression levels of GAP-43 and GFAP among the H, UT, and T groups. The T group demonstrated relatively better preservation of GAP-43-positive neuronal fibers and higher GFAP levels compared to the UT group, highlighting the neuroprotective and neuroregenerative effects of MLC901 (Fig. 6a, b). The T group showed significantly improved functional recovery and exhibited better motor coordination, faster sensory withdrawal response, and improved nerve conduction compared to UT. Reduced tissue damage, smaller hemorrhagic foci, and less albumin leakage were observed in the T group. Hence, it can be stated that MLC901 T promoted neuronal survival and axonal regeneration, suggesting its potential as a therapeutic agent for SCI recovery.

## Discussion

This study investigates the efficacy of MLC901 in promoting functional recovery and improving sensory, electrophysiological, neurological, and histological outcomes following KA-induced SCI. To ensure that the impairments observed in Fig. 2 are due to the KA and not a potential injury caused by the intrathecal injection, we conducted a pilot study in which we compared the sham group undergoing surgery without KA injection to healthy control, and no significant difference was observed between the sham and H groups, so we included the sham group as H animals that underwent same laminectomy surgery but receive an equal volume of normal saline. Our findings highlight the potential of MLC901 as a therapeutic agent for SCI, demonstrating its neuroprotective and neuroregenerative effects across multiple assessments, including locomotor, sensory, and electrophysiological recovery. The underlying mechanisms by which MLC901 is attributed to its multi-targeted neuroprotective properties, particularly in response to excitotoxic damage induced by KA. KA, a potent glutamate receptor agonist, initiates a cascade of events leading to excitotoxicity, oxidative stress, and, ultimately, neuronal apoptosis. By modulating glutamate receptor activity, MLC901 may mitigate excitotoxic damage and preserve neuronal integrity [23]. Previous studies have indicated that MLC901, through its anti-inflammatory, antioxidant, and neurotrophic effects, can improve functional recovery after SCI [7, 20, 24]. Our previous study also demonstrated that KA induces significant neurodegeneration and apoptosis in motor neurons, leading to substantial cell damage. This damage is characterized by reduced cell viability, downregulation of key regenerative markers such as AKT, p-AKT, and GAP43, and upregulation of the apoptotic marker p-GSK3β, highlighting the detrimental effects of KA on motor neurons [7].

Locomotor recovery, as assessed through the BBB scale, revealed significant improvements in hindlimb coordination and motor function in MLC901-T rats. These results align with previous studies showing that treatments targeting excitotoxicity can enhance locomotor recovery, albeit full functional recovery remains challenging [25]. The significant improvements in jaw movement, paw placement, and toe clearance suggest that MLC901 treatment positively affects multiple aspects of motor control, likely through neuroprotection and enhanced neuroplasticity. This is in line with the findings of our previous studies of the mechanical scratch injury model in mature motor neurons and a calibrated forceps compression SCI model in SD rats, followed by MLC901 treatment. MLC901 significantly improved cell survival and regeneration of the mature motor neurons, improved hind-limb motor function, alleviated pathological damage, and enhanced locomotor activity in SD rats. These effects were associated with the activation of the PI3 K/AKT/GSK-3β pathway, suggesting that MLC901 mitigates apoptosis, promotes neurogenesis, and aids functional recovery post-SCI [16, 24]. A similar result was also reported by another study, in which MLC901 T Wister rats significantly improved motor function and histopathological outcomes after chronic severe SCI by reducing NG2 and Caspase-3 expression while increasing IL-10 levels. These effects were attributed to the suppression of gliosis, neuroapoptosis, and neuroinflammation and enhancing overall recovery [17].

The running wheel test assesses hind limb strength and coordination by evaluating the rats’ ability to maintain and control the running wheel [26, 27]. The inverted grid test evaluated the strength and coordination of both hindlimbs and forelimbs by measuring the ability of the rats to hold onto a grid that had been turned upside down. This result showed that MLC901 treatment positively impacts motor coordination and strength recovery, suggesting better motor function and coordination as indicated previously [28, 29]. The sensory recovery observed through the thermal response test further supports the notion that MLC901 promotes the restoration of sensory function after SCI. Sensory loss is a critical aspect of SCI recovery, and the enhanced withdrawal latency observed in MLC901-T rats indicates improved sensory nerve conduction. This finding is consistent with prior work showing that neuroprotective agents can enhance sensory function recovery, which is crucial for overall rehabilitation [28]. MLC901 was reported to also influence neuropathic pain by modulating miR30c-5p, TGF-β, and VEGF receptor expression; MLC901 promoted axon demyelination and reduced neuropathic pain behavior, offering potential targets for further research into therapeutic interventions [30].

Electrophysiological analysis through somatosensory evoked potentials (SEPs) provided valuable insights into neural activity and conduction following SCI [31, 32]. MLC901 treatment improved SEP amplitude, suggesting enhanced neural function and transmission. Previous research has highlighted the significance of SEP recovery as an indicator of neural regeneration following SCI [33]. The statistically significant difference in amplitude and duration between the groups suggests that MLC901 enhances neural transmission and possibly facilitates the repair of damaged pathways, an important consideration in recovery strategies for spinal cord injuries [24]. The recovery of SEP amplitude in the MLC901 T rats indicates that the compound may aid in restoring damaged neural circuits, a key factor for functional recovery after SCI.

The morphological and pathological analysis provides crucial insights into the structural changes in the spinal cord following SCI and the effects of therapeutic interventions [34]. Histological and immunohistochemical analyses further underscore the neuroprotective effects of MLC901. The reduced neuronal loss, smaller lesion areas, and less pronounced hemorrhagic foci in T rats demonstrate the compound’s ability to mitigate secondary injury processes, such as blood–brain barrier disruption and neuronal apoptosis. MLC901’s ability to promote neuronal survival is evidenced by the enhanced expression of GAP-43, a marker of neuronal regeneration. Moreover, the preservation of GFAP-positive astrocytes in the T rats highlights the supportive role of astrocytes in neuroprotection and repair. Astrocytes contribute to neuronal survival by maintaining homeostasis, providing metabolic support, and secreting neurotrophic factors [35]. The reduced astrocyte loss in MLC901 T rats suggests that MLC901 helps maintain a favorable environment for neuronal recovery and regeneration.

The rationale for using MLC901 in this study is rooted in its herbal composition, which has been shown to possess anti-inflammatory, neuroprotective, and neuroregenerative properties. MLC901 has previously demonstrated efficacy in various models of neurodegenerative diseases, including stroke and Alzheimer’s disease, where it facilitated functional recovery and reduced neuronal damage [29]. Given its multi-targeted action, MLC901 is a promising candidate for treating SCI, where multiple pathways contribute to the progression of injury and impairment. By addressing both the excitotoxic damage caused by glutamate and the inflammatory processes that follow SCI, MLC901 may offer a holistic approach to SCI treatment.

Although the current study provides compelling evidence of the neuroprotective and neuroregenerative effects of MLC901, there are several limitations to consider. First, further studies are needed to elucidate the precise molecular mechanisms underlying MLC901’s effects. Investigating how MLC901 interacts with glutamate receptors, inflammatory mediators, and neurotrophic pathways will provide a clearer understanding of its therapeutic potential. A limitation of the current study is the lack of direct assessment of macrophage and microglia involvement in the inflammatory response. While the study suggests an inflammatory response reduction, further analysis using specific markers for macrophages and microglia, such as IBA1, TMEM117, or F4/80, is recommended for confirming the presence and extent of inflammation. Additionally, studies evaluating the long-term effects of MLC901 in chronic injury models are essential to assess its efficacy during later stages of SCI, as well as any potential side effects or limitations of prolonged use. Finally, dose–response relationships and the impact of varying KA concentrations on injury severity should be explored to optimize experimental conditions and treatment protocols.

## Conclusion

The findings of this study robustly demonstrate the successful induction of spinal cord injury (SCI) through KA intraspinal administration and underscore the efficacy of MLC901 as a neuroprotective and neuroregenerative agent. The study effectively modeled incomplete paraplegia in rats and highlighted the impact of SCI on various physiological and pathological outcomes. The intraspinal administration of KA successfully induced SCI, leading to notable functional impairments, including incomplete paraplegia. This model effectively replicates the secondary injury phase following SCI, characterized by significant motor and sensory deficits. A concentration of MLC901 at 10 mg/kg/day was shown to be effective in ameliorating the adverse effects of SCI. The treatment resulted in significant improvements across various assessment parameters, including locomotor and sensory functions. MLC901 T rats exhibited enhanced performance in the open field test, running wheel test, inverted grid test, and grid walk test. These improvements reflect better motor coordination, strength, and overall functional recovery. Electrophysiological recordings revealed improved nerve conduction in MLC901 T rats, indicating effective mitigation of neuronal damage and enhanced axonal regeneration. Histological analyses showed reduced pathological damage and improved tissue preservation in the MLC901 T group. Additionally, increased expression of regenerative markers such as GAP43 and GFAP further supported the neuroprotective and neuroregenerative effects of MLC901. Overall, MLC901 demonstrates substantial promise in promoting recovery and protecting against secondary injury effects in spinal cord injury models. The comprehensive improvement across multiple assessment domains validates its efficacy and supports its potential for further clinical exploration.

## Methods

### KA Administration

All experimental procedures were approved by the University Kebangsaan Malaysia Animal Ethical Committee (UKMAEC) under approval number TEC/FP/2021/YOGESWARAN/27-MAY/1176-JUNE-2021-JUNE-2023. Detailed methods for KA injury have been previously published in our protocol paper [5]. Briefly, fifteen adult Sprague–Dawley rats (weighing 300–400 g) were sourced from the Specific Pathogen Free (SPF) facility at the Animal Experimental Unit (AEU), Faculty of Medicine, University Malaya, Malaysia. The rats were housed in clean cages with a bio-bubble air control system, maintained on a 12-h light–dark cycle, and provided with ad libitum access to food and water. They were acclimatized for 7 days before the experiment. Pre-injury, the rats were randomly assigned to one of three groups (*n* = 5/group): T, UT, and H. Post-injury rats were housed individually in cages. KA was administered intrathecally at a dose of 0.05 mM as a 10 µL/100 g body weight injection in normal saline at a rate of 10 µL/min by intra-spinal injection between the T12 and T13 thoracic vertebrae. While the H rats got 40 µL of saline solution at the slow rate of 10 µL/min, no physical damage was observed in H rats. Rats in the T group were administered NeuroAiD (MLC901) orally at a dose of 10 mg/kg/day for 28 days, mixed in their drinking water.

### Experimental Design

The experimental timeline and assessment schedule are illustrated in Fig. 1. Pre-assessments were conducted on days − 7, − 1, 1, 3, and 7. Following the surgery, electrophysiological evaluations were performed on days 0, 14, and 28. Locomotor assessments were conducted on days 3, 7, 14, 21, and 28, while sensory function tests were carried out on days 7 and 21. On day 29, the animals were euthanized by intraperitoneal administration of 2 mL of pentobarbital (200 mg/mL), and spinal cord specimens were collected for histological and pathological analysis. The details about locomotor, electrophysiology, and pathological assessments have been explained in the Supplementary data file. The flow diagram of the study is also presented as Figure S1.

### Locomotion and Behavioral Tests

#### Open Field Test (OFT)

The OFT was employed to evaluate changes in locomotor activity following our previously established protocol [36]. Rats were positioned in the center of an acrylic box, where their locomotor behavior was observed for 5 min by two blinded observers. The open field was divided into three concentric zones: an inner square (20 cm), a middle square (40 cm), and an outer square (60 cm), with the floor marked by 10 cm × 10 cm grids. The number of boxes crossed by each rat within the 5-min observation period was recorded on days 3, 7, 14, 21, and 28 after the injuries.

#### Running Wheel Test

The running wheel test was used to evaluate motor deficits. Rats were placed on a wheel that was forcibly rotated 90°. The time taken for the rats to stop the wheel by coordinating their forelimbs and hindlimbs was recorded. The following scoring scale was applied, as described previously [37].

**Table:** 

0	No restraining
1	Poor restraining
2	Slight restraining
3	Moderate restraining
4	Complete restraining

#### Grid Walk Test

An elevated metal grid (40 × 60 cm) was utilized for the test. After cleaning the grid and apparatus with 70% ethanol, rats were placed at one end and allowed to walk to the other end. Their walking behavior was recorded, including the number of foot faults, total footsteps, and the time taken to cross the grid [38]. Data analysis was performed by an experimenter who was blinded to the experimental groups.

#### Inverted Grid Test

To assess muscle strength, rats were placed on an inverted grid positioned approximately 20 cm above the ground. The maximum time each rat could hold onto the grid was recorded [29].

### Sensory Function Tests

#### Hot Spatula Method

Sensory nociception was assessed using a hot spatula. A standard temperature was established by placing the hot spatula on the rat’s tail and gradually increasing the temperature until the rat began licking its tail [39]. This temperature served as the baseline. The latency to response was recorded and compared across groups. The procedure was repeated 3–5 times, and the mean values were used as threshold values. Scoring was based on the withdrawal response, evaluated by using the scale mentioned below.

**Table:** 

0	No withdrawal
1	Slow withdrawal
2	Prolonged withdrawal
3	Quick withdrawal and repeated flicking

#### Cold Sensation Test

Cold sensation was tested using 98% absolute alcohol. The area of the skin to be tested was shaved, and alcohol was sprayed onto it [39]. The response was recorded and graded using the same scale as the hot spatula method.

### Electrophysiological Assessment

#### Somatosensory Evoked Potentials (SEPs)

SEPs were measured using a Nicolet® Viking Quest™ system (USA). Rats were anesthetized with a 9:1 mixture of Ketamine and Xylazine. Stimulus electrodes were placed on the hind legs, and recording electrodes were positioned over the hind-limb cortical sensory area. Reference electrodes were placed 0.5 cm posterior to the recording electrodes. SEPs were recorded using a direct-current square wave stimulus (10–30 mA, 0.1 ms pulse width, 1 Hz frequency). Changes in latency, duration, and amplitude were recorded following the previously established protocol [31, 40].

### Histological Analysis

Four weeks post-surgery, rats were euthanized using pentobarbital (200 mg/mL). Spinal cord tissues (T12-T13) were fixed overnight in 4% paraformaldehyde (PFA) and then sectioned into 15 µm thick sagittal and parasagittal slices using a cryostat. Hematoxylin and eosin staining were performed for general histological examination, which was conducted using a light microscope (Olympus, Tokyo, Japan).

### Immunohistochemical Analysis

Immunohistochemistry was performed on 5-µm thick tissue slices. The sections were incubated overnight at 4 °C with monoclonal GAP43 (D9 C8) Rabbit antibody (1:200, Cell Signaling, USA) and GFAP Monoclonal Antibody (1:200, ThermoFisher Scientific). Following incubation, the sections were stained with anti-rabbit IgG Alexa 488 and Alexa 594 secondary antibodies (1:400, Santa Cruz Biotechnology) at 37 °C for 2 h. Nuclear staining was carried out with DAPI (1:15,000) in DPBS for 30–40 min. The slides were mounted and examined using a confocal microscope (Nikon A1R).

### Statistical Analysis

Statistical analysis was conducted using SPSS version 19.0 software (IBM Corp., Armonk, NY, USA) and GraphPad Prism 10. Results are presented as mean ± standard error of the mean (SEM), with specific values provided in each figure legend. The symbol n denotes the number of animals used and analyzed. For comparisons between two independent groups, parametric values were assessed using Student’s *t*-test. For multiple comparisons, a one-way analysis of variance (ANOVA) followed by Dunnett’s post-hoc test was employed. Statistical significance was set at *p* < 0.05, *p* < 0.01, and *p* < 0.001.

## Supplementary Information

Below is the link to the electronic supplementary material.

Supplementary file1 (DOCX 1841 KB)

## Data Availability

No datasets were generated or analysed during the current study.

## Declarations

### Patents

NeuroAid II (MLC 901) as Spinal cord Regeneration therapy.
