# Supplementary material for: Evaluation of Neuroprotective and Neuroregenerative Potential of NeuroAiD™ II(MLC901) in a Rat Model of Kainic Acid-Induced Spinal Cord Injury
Source: Mol Neurobiol. 2025 Jun 6;62(10):13060–72. doi: 10.1007/s12035-025-05064-4 (PMC12433347; doi:10.1007/s12035-025-05064-4)
Supplement: Supplementary file 1 — Supplementary file1 (DOCX 1841 KB) [file 12035_2025_5064_MOESM1_ESM.docx]

**Supplementary Data: Figures**


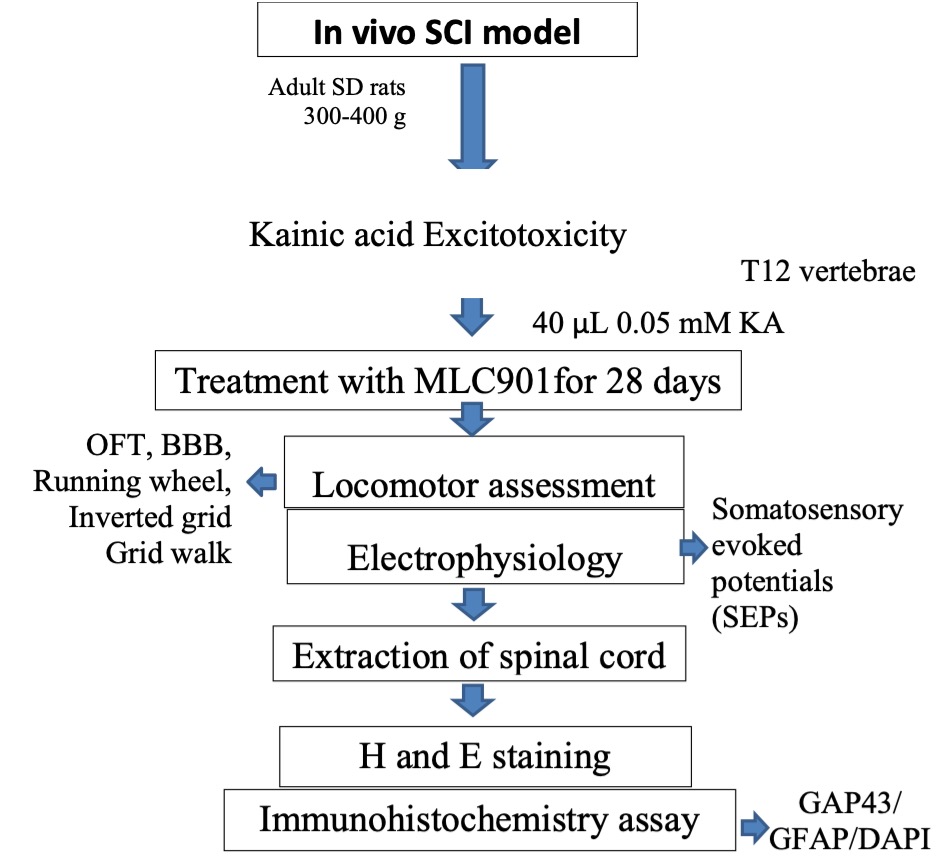


**Figure S1**: Flow diagram explaining the KA excitotoxicity injury in adult SD rats following MLC901 treatment and neuroprotection.


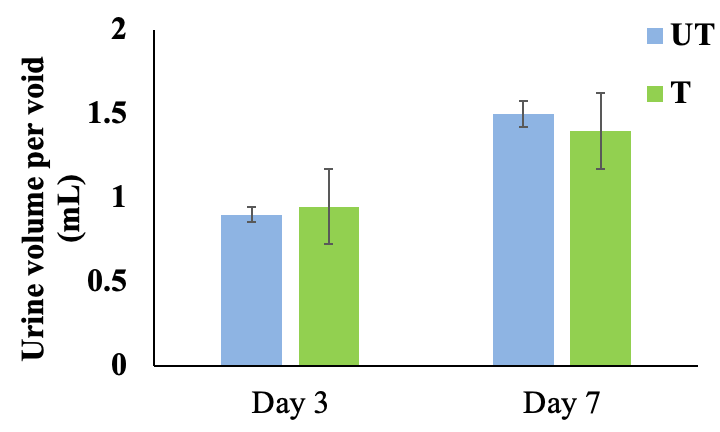


**Figure S2:** Urine volume per void: Manual bladder voiding by the experimenter was required for 1 week (Day 7) post-SCI, after which spontaneous recovery began. No significant difference was observed between UT and T rats. Data were analyzed using Student’s *t*-test for non-parametric and unpaired comparisons (*n* = 6).


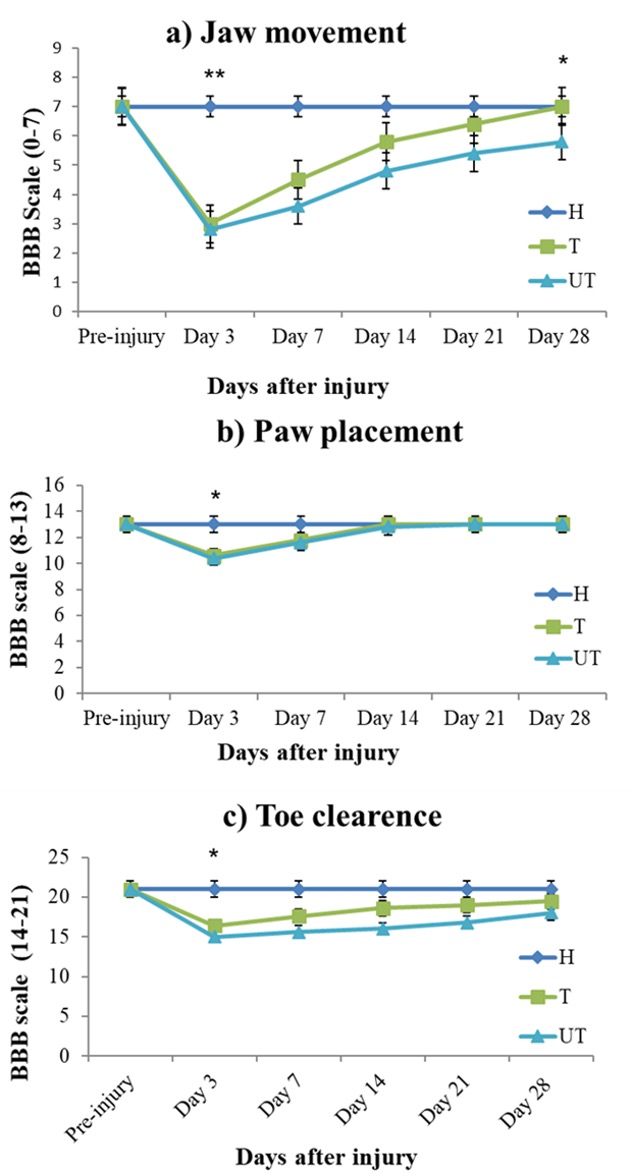


**Figure S3**: BBB Subcategories: The BBB score was further divided into three subcategories for a more detailed evaluation: jaw movement (0-7) (a), paw placement (8-13) (b), and toe clearance (14-21) (c). The results showed that T rats scored significantly better in jaw movement, paw placement, and toe clearance compared to UT rats by day 28 (p < 0.05*). Statistically significant differences were also noted in each sub-score category when comparing T and UT rats to H rats (p < 0.01 and p < 0.05), as shown in Figures a, b, and c.


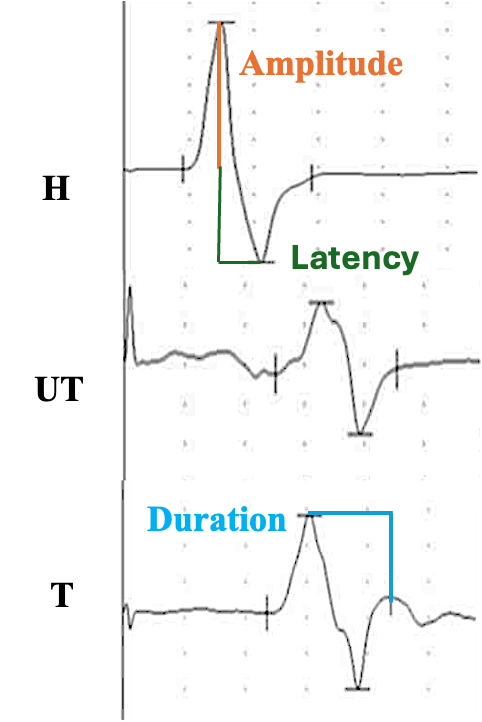


**Figure S4:** The representative SEPs waveforms of different groups, where H) Sham or Healthy control, UT) are untreated rats after KA injury and T) MLC901 treated rats after 14 days post-injury.


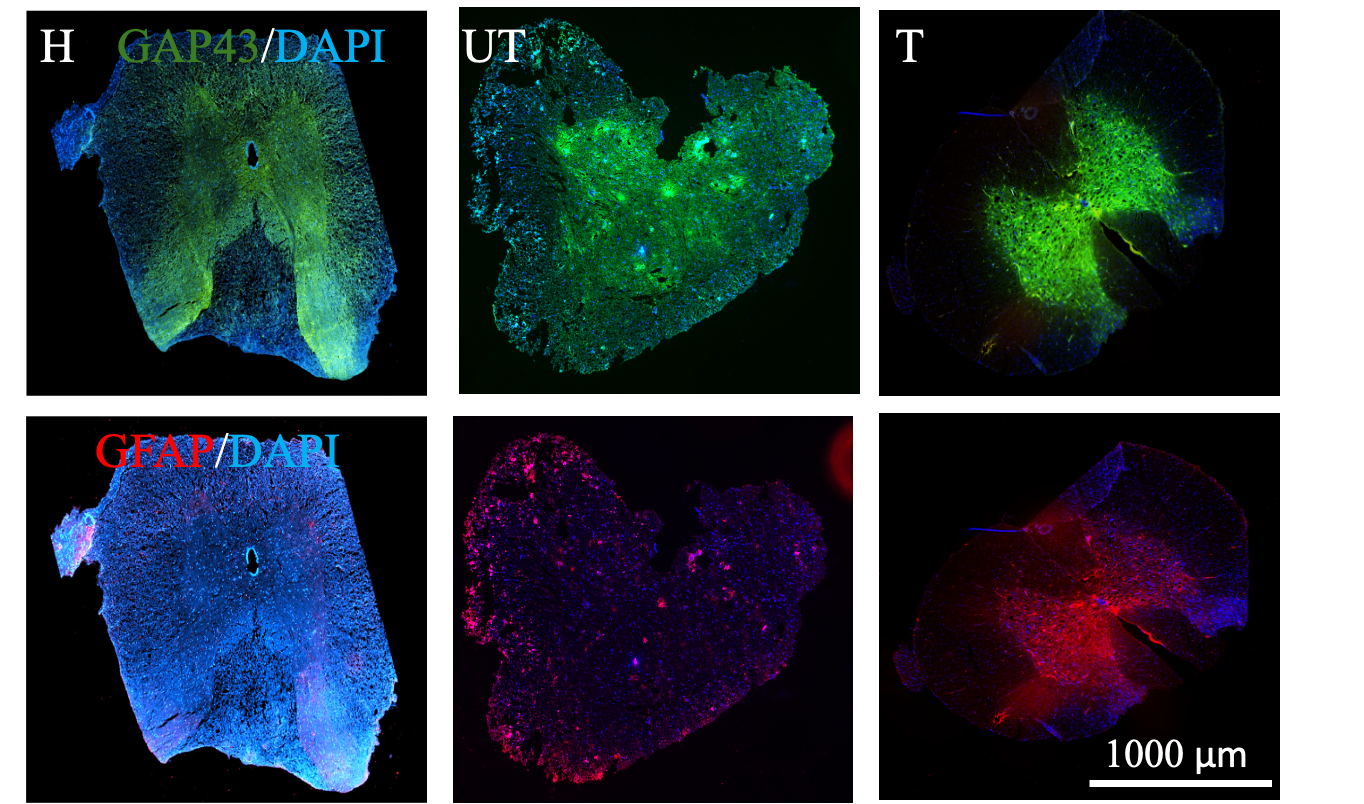


**Figure S5:** Immunohistochemical Analysis showing co-staining of GAP-43/DAPI, GFAP/DAPI expression in H, UT, and T groups.
